# Gualou Guizhi decoction reverses brain damage with cerebral ischemic stroke, multi-component directed multi-target to screen calcium-overload inhibitors using combination of molecular docking and protein–protein docking

**DOI:** 10.1080/14756366.2017.1396457

**Published:** 2017-11-29

**Authors:** Juan Hu, Wen-Sheng Pang, Jing Han, Kuan Zhang, Ji-Zhou Zhang, Li-Dian Chen

**Affiliations:** a Fujian Academy of Traditional Chinese Medicine, Fuzhou, PR China;; b School of Rehabilitation Medicine, Fujian University of Traditional Chinese Medicine, Fuzhou, PR China;; c The Second People’s Hospital of Fujian Province, Fuzhou, PR China

**Keywords:** Gualou Guizhi decoction, reverses brain damage, calcium-overload, inhibitors screening

## Abstract

Stroke is a disease of the leading causes of mortality and disability across the world, but the benefits of drugs curative effects look less compelling, intracellular calcium overload is considered to be a key pathologic factor for ischemic stroke. Gualou Guizhi decoction (GLGZD), a classical Chinese medicine compound prescription, it has been used to human clinical therapy of sequela of cerebral ischemia stroke for 10 years. This work investigated the GLGZD improved prescription against intracellular calcium overload could decreased the concentration of [Ca^2+^]*_i_* in cortex and striatum neurone of MCAO rats. GLGZD contains Trichosanthin and various small molecular that they are the potential active ingredients directed against NR2A, NR2B, FKBP12 and Calnodulin target proteins/enzyme have been screened by computer simulation. “Multicomponent systems” is capable to create pharmacological superposition effects. The Chinese medicine compound prescriptions could be considered as promising sources of candidates for discovery new agents.

## Introduction

Stroke is a disease of the leading causes of mortality and disability across the world. The World Health Organization (WHO) places the global occurrence of cerebral ischemia at around 200 cases per 100,000 inhabitants, although the data varies among countries^1^. One of its main types is cerebral ischemia stroke, caused by the obstruction of cerebral vessel which leads to reduction of blood flow to the brain. Despite intra-arterial thrombolysis, neuroprotective agents, anticoagulant, antiplatelet, as well as vasodilatory agents have been widely used in clinical to treat the stroke^2^, but the benefits of curative effects look less compelling; side effects from these drugs are common.

Ischaemic stroke was caused by the obstruction of cerebral vessel and a subsequent reduction in the chemical adenosine triphosphate (ATP), often damaging brain beyond repair. ATP can provide energy for cell activity; this energy loss leads to impaired cellular function due to reduced ATP-dependent processes and a disruption in ionic gradients across membranes. In such the case, there is a significant efflux of potassium ion (K^+^) and the movement of extracellular calcium into cells through calcium channels. This increment in intracellular calcium that leads to the “calcium toxicity,” that is “calcium overload.” Intracellular calcium triggers the break-down of phospholipids, proteins and nucleic acids and contributes to structural and functional damage of the cell membrane, which facilitates cell death. Intracellular calcium overload is considered to be a key pathologic factor for ischemic stroke^3^^,^^4^. Calcium channel blockers would be as potential candidates for the treatment of stroke disease. However, the intracellular calcium overload from stroke is complex: deferent drugs that modulate deferent target can only enable a palliative treatment instead of curing or repairing the nerve injury. At present, the some number biological targets for therapeutics have been identified included N-methyl-D-aspartate receptors NR2A and NR2B subunit proteins, calcineurin enzyme (CaN), and calmodulin protein (CaM). Furthermore, a number of antagonists such as 1N4, ifenprodil, FK-506, and trifluoperazine which has been used treatment with stroke disease, but their clinically efficacy was limited.

Traditional Chinese medicine (TCM), a system that dates back more than 2000 years, it plays a great role in the Chinese health care system^5^. Herbals against stroke which includes plant derived phytochemicals, single plant crude extracts and compound formulations. Gualou Guizhi decoction (GLGZD), a classical prescription of TCM that consisting of six herbs as: the root of *Trichosanthes kirilowii* Maxim. (Tianhuafen), *Cinnamomum cassia* Presl (Guizhi), *Paeonia lactiflora* Pall (Baishao), *Zingiber officinale* Rose (Ganjiang), *Glycyrrhiza uralensis* Fisch (Gancao), and *Ziziphus jujuba* Mill (Dazao). This research group used GLGZD to human clinical therapy of sequela of cerebral ischemia stroke for 10 years. GLGZD modified Barthel index and myoelectricity activities of facial electrogram in the treated group improved better than those in the control group results suggest that had significant efficacy on treating limbs spasm from cerebral apoplexy^6^. Previous studies have reported that GLGZD can improve middle cerebral artery occlusion (MCAO) rat symptoms via inhibiting over-activation of astrocytes would controlling apoptosis of neurons and up-regulation of neuronal specific MAP-2 and NeuN markers. GLGZD might be a potential neuroprotective agent for stroke^7^. During the reperfusion after MCAO rat, GLGZD could significantly decrease the content of MDA and increase the activity of SOD, GSH-PX, and CAT. The protective mechanism of GLGZD on focal cerebral ischemia injury may be related to restrain the lipid per oxidation, promote removal of oxygen free radicals, compete with oxygen-free radical injury, and protect neural cells from injured^8^.

There are many active ingredients in the TCM formula which is the potential sources of lead compounds for drug formulations. TCM greatest advantage is bioactive ingredients synergism by such network-based. A traditional Chinese prescription is a complicated multilevel system including numbers and species of the substances, drug pair compatibilities, diversity and interdependence, etc. The complicated system that is does not only lend simple experimental to be the perfect solution. Computer simulation technique being used, the complicated problem was solved quickly and accurately^9–12^. In this article, we are going to examine the effect of pharmacological intervention by observing the changes of intracellular Ca^2+^ concentration in cerebral cortex and striatum and to explore the possible mechanism by which GLGZD repairs the nerve injury on MCAO model rat. Based on above pharmacophores, the information of 248 ingredients in GLGZD collected from electronic search engines such as PubMed, Baidu Scholar, Springer, Science Direct; Chinese medical literatures and academic publications, as well as TCM database; it is used to screen anti calcium overload inhibitors. According to one-component to multi-targets and multi-components to multi-targets modes, CaN, CaM, NR2A, and NR2B inhibitory activities by molecular docking or protein–protein docking assessments of compounds. It will be looked forward to the exploration of its theories and set the stage for clinical treatment.

## Materials and methods

### Chemicals and agents kit

DMSO was purchased from Sigma-Aldrich (Shanghai, China). An aliquot of 0.25% Tripsin-EDTA, Trypan blue, and PBS (calcium and magnesium free) were purchased from Nanjing KeyGen Biotech. Co. Ltd. (Nanjing, China). GIBCO DMEM was produced by Invitrogen (Grand Island, NY). Fluo-3 AM (Calcium ion fluorescence probe, 5 mM) was purchased from Beyotime Institute of Biotechnology (Haimen, China). Water was deionised using the Milli-Q-Plus ultra-pure water system (Millipore, Milford, MA).

### Chinese medicines sliced and GLGZD

Medicinal slices of TCM material, the root of *Trichosanthes kirilowii* Maxim. (Tianhuafen), *Cinnamomum cassia* Presl (Guizhi), *Paeonia lactiflora* Pall. (Baishao), *Zingiber officinale* Rose. (Ganjiang), *Glycyrrhiz uralensis* Fisch. (Gancao), and *ZiziPhus jujuba* Mill. (Dazao), were purchased from Fujian Xiang’an Pharmaceutical Co., Ltd. (Quanzhou, China). Herbal remedies accorded with the 2015 edition Chinese pharmacopoeia standard after testing^13^. Six Chinese medicines sliced mixture in prescription proportion was crushed to powder (60 smash), added six times cold-water to soak in 30 min and let it simmer 30 min, strain the medicine juice through a piece of gauze, every boil the volume of decoction about be 150 ml; repeat again. The first filtrate was merged with the second filtrate, was concentrated in a rotary evaporator until the liquid has reduced to 120 ml.

### GLGZD fingerprint analysis by UPLC

Shimadzu UFLC-XR system was performed with model LC-20AD XR prominence LIQUID CHROMATOGRAPH pump, DGU-20A3 prominence degasser, SIL-20 A XR prominence auto sampler, CTO-20 A prominence column oven, as well as SPD-M20A prominence diode array detector. The mobile phase was acetonitrile (A) − 0.5% formic acid aqueous solution (B). The flow rate was 0.5 ml/min, the column temperature sets 40 °C, detection wavelength at 254 nm, sample size 5 μl.

### Middle cerebral artery occlusion (MCAO) rat model

Adult male Sprague Dawley rat (230 ± 20) g. Animals were fasted before 12 h, free drinking water. Cut a 4–0 nylon suture into 20 mm segments. Ten percent chloral hydrate was used to tranquilize Rat. Make a 1 cm long midline incision on the rat neck. Use retractors to expose the surgical field and identify the right common carotid artery (CCA), external carotid artery (ECA), and internal carotid artery (ICA). Carefully dissect the arteries free from surrounding nerves and fascia. Dissect the ECA further distally and coagulate the ECA and its superior thyroid artery (STA) branch using a bipolar coagulator. Cut the ECA and STA at the coagulated segment. Loosely tie two sutures around the ECA stump. Apply a vascular clamp at the bifurcation of the CCA into the ECA and ICA. Make a small incision at the end of ECA stump with scissors. Insert the suture into the incision and advance to the clamp. Tighten the two silk sutures around the lumen just enough to secure yet preserve mobility of the in-dwelling monofilament suture. Remove the clamp from the bifurcation. Gently advance the monofilament suture from the lumen of the ECA into the ICA for a distance of 9–10 mm beyond the bifurcation of CCA to occlude the origin of MCA. The duration of surgery is about 30–45 min. Suture the incision on the neck and returns it to the cage. To perform transient MCAO, after 2 h, the researcher anesthetised rat again with a half dose of chloral hydrate, and withdraw the suture back into the stump of ECA, reperfusion 24 h. In the sham operation group, except for inserting wires, the remaining operation was the same as the ischemia-reperfusion model group^14^.

The project was authorised by Fujian Provincial Department of Science & Technology (2013Y0047) and approved by the Fujian Academy of Traditional Chinese Medicine ethical committee for animal studies (FJATCM-IAEC 2015009). The procedures agree with the international rules for animal safety.

### Experimental grouping

Five groups of SD rats, ten in each received the following treatment schedule. A dose of medicine needs to be boiled concentrated in volume of decoction is limited to 120 ml, rats were treated with GLGZD according to human clinical dosage converted into rat oral administration, 10 ml/kg, for 7 d. MCAO rats model were prepared.

Group I: Sham operated group (SO).

Group II: Middle cerebral artery occlusion group (MCAO).

Group III: GLGZD classical prescription (GLGZD-CP), a dose of medicine contained Tianhuafen 6 g, Guizhi 9 g, Baishao 9 g, Ganjiang 9 g, Gancao 6 g, and Dazao 12 grains.

Group IV: GLGZD improved prescription (GLGZD-IP), only different amount of Tianhuafen, the other herbs were the same as classical prescription, 30 g Tianhuafen instead of 6 g.

Group V: Tianhuafen group (THF), Tianhuafen 30 g.

### Repaid separation of cerebral cortex and striatum nerve cell of rats

Rats were anesthetised by 10% chloral hydrate after establishing the model and the cerebrum were obtained from the cranium^15^. The cortex and striatum were separated under the condition of iced bath and were put into pre-cooling DMEM nutrient medium. Then, the cell suspension was immediately made. The neuron suspension separated was put in to centrifuge tube with the volume of 2 ml. The number of cell in each centrifuge tube was a proximately 1 × 10^7^.

### Determination of free [Ca^+^] in the neurone

Fura-3/AM (final concentration was 5 µmol/l) was put into cell suspension (1 ml) and was agitated at constant temperature of 37 °C. The cells were washed twice time with Hank’ solution. The final cell concentration was modulated to 1 × 10^6^/ml. The fluorescence intensity of single cell was randomly determined with the Thermo Scientific Varioskan Flash spectral scanning multimode reader combines fluorescence intensity, time-resolved fluorescence, photometric, and optional luminometric detection technologies (excitation wavelength 488 nm and emission wavelength the 530 nm)^15^.

### Molecular protein/enzyme docking

Molecular docking was performed using the programmed eHiTS version 12 from SimBioSys Inc (http://www.simbiosys.com/ehits). This is an exhaustive flexible docking algorithm with a scoring function which incorporates both empirical and knowledge-based features, and applies an exhaustive conformational search, an automated protonation state handling, and a tunable scoring function for the ligands and targets^16^.

The retrieval of chemical components of TCM database, in GLGZD, Tianhuafen, Guizhi, Baishao, Ganjiang, Gancao, and Dazao, six herbs, a total of 248 small molecule compounds. Draw its chemical structure and output it in Mol and Sdf files.

The crystal structures of NR2A (PDB ID 4JWX) in complex with a threonine-to-alanine point mutation in the NR2A ligand binding site competitive manner agent 1N4^17^, NR2B (PDB ID 3QEL) with small molecule inhibitor ifenprodil^18^, FKBP12 (PDB ID 4DH0) with inhibitor FK506^19^, and Calmodulin (PDB ID 1A29) with inhibitor trifluoperazine^20^ in Protein Data Bank was selected for the docking study. The eHiTS software package was used for flexible docking. Active site detection was carried out using the “-complex” parameter. Each docking procedure must be validated with a blind docking with the experimental ligand of the protein/enzyme target; the root mean square deviation (RMSD) between experimental and calculated structures should be less than 2.0. The compound was then docked into the active site using the highest accuracy mode of docking (“-accuracy” parameter set to 6). The programme automatically detected the ligand in the complex and selected the part of target protein within a 7 Å margin around the ligand to be the active site. The scoring was according to the eHiTS score that is included in the eHiTS software package. We selected the compound with the best score and speculated the detail binding patterns^21^.

### Protein–protein docking

Tanhuafen is rich in protein, Trichosanthin (TCS). The crystal structure of TCS in Protein Data Bank ID is 1TCS. In order to understand the important residues interaction with proteins/enzyme, the docking of TCS domain with the FKBP12, Calmodulin NR2A and NR2B were carried out. The ZDOCK docking programmes was used for protein–protein docking simulations with default parameters^22^. A total of 3600 structural conformations were generated for every docking, and the best conformation was evaluated by binding mode analysis. The resulting docked complexes from both docking programs were analysed to understand interactions of TCS amino acid residues with target proteins or enzyme.

## Results and discussion

### Phytochemical characterisation of GLGZD (fingerprint by UPLC)

Chromatographic separation was achieved on a Shim-pack XR ODS II (2.2 μm × 75 × 2.0 mm) column was produced by Shimadzu Corporation (Kyoto, Japan). The gradient programme of the mixed mobile phase ware listed in Table 1.

**Table 1.** The gradient elution programme of the UPLC.

Time (min)	A% (acetonitrile)	B% (0.03% phosphoric acid)
0	5	95
5	8	92
8	8	92
10	10	90
16	15	85
19	17	83
20	17	83
27	30	70
37	40	60
40	60	40
50	90	10

**Figure 1.**
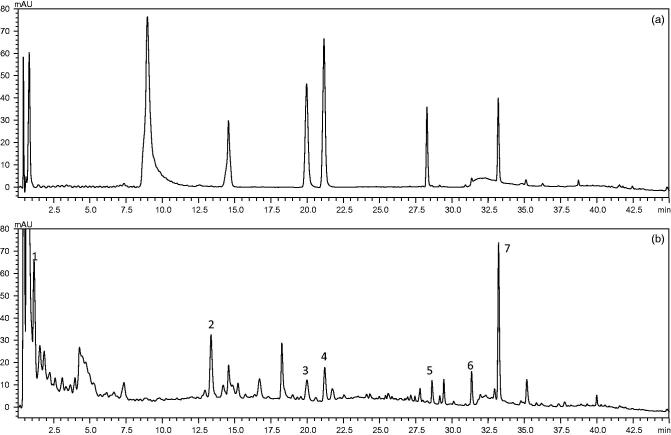
The UPLC chromatographic fingerprint of GLGZD, (a) reference substance chromatographic fingerprint, (b) GLGZD chromatographic fingerprint. Seven peaks were labelled and the name of the compound represented by each peak numbered from 1 to 7 as gallic acid, paeoniflorin, liquiritin apioside, cinnamic acid, onospin, isoliquiritigenin, and glycyrrhizic acid, respectively.

Construction of the chromatographic fingerprints plays an important role in the multi-component separation and determination of complex. Here, the authors optimise the key determine parameters for the compounds establish an UPLC chromatographic fingerprint of GLGZD. Discriminating the characteristic peaks of had the best responses at 254 nm, especially with the retention time of 5–60 min in which seven major components showed the greater absorbance (Figure 1). Contrasted with reference substance, they were differentiated with UPLC method. The name of the component represented by each peak numbered from 1 to 7 as gallic acid, paeoniflorin, liquiritin apioside, cinnamic acid, onospin, isoliquiritigenin, and glycyrrhizic acid. Detected GLGZD for seven major components content was 5.38, 3.59, 1.33, 2.61, 2.03, 2.87, and 8.74 mg/g, respectively.

### Effect of GLGZD on [Ca^2+^]_i_ in cerebral cortex and striatum neurone of MCAO rats

**Figure 2.**
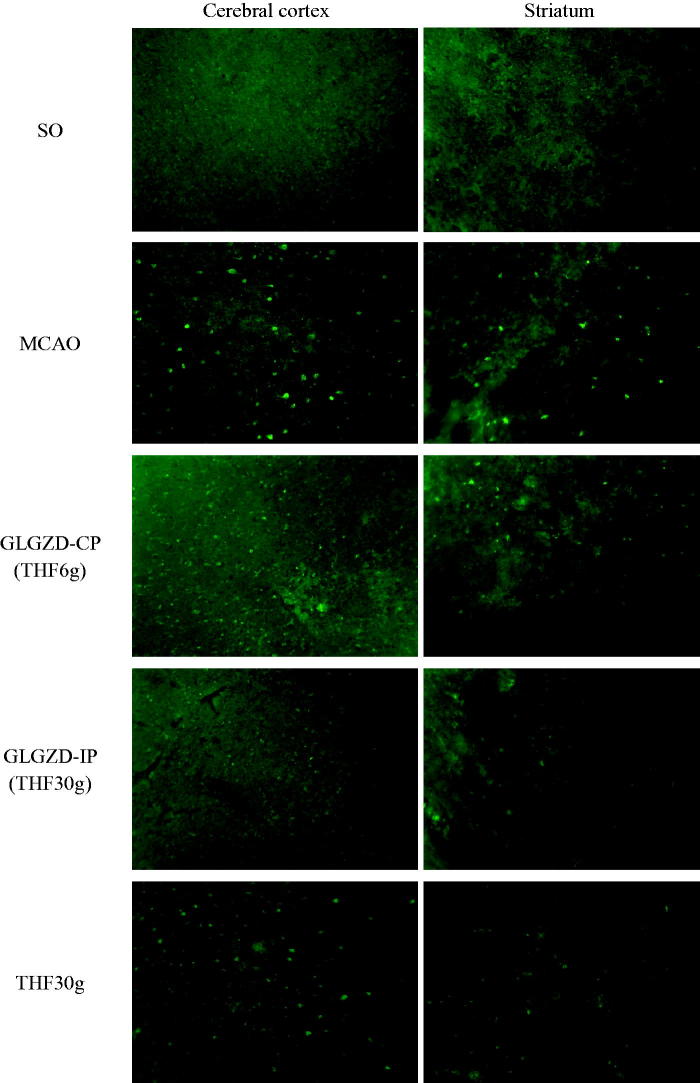
Fluorescence micrographs of [Ca^2+^]*_i_* in cerebral cortex and striatum neurone of MCAO rats (100×).

The mean fluorescence of intracellular free calcium ([Ca^2+^]*_i_*) in cortex and striatum neurone of MCAO group significantly increased was compared with SO group. The mean fluorescence of [Ca^2+^]*_i_* in cortex and striatum neurone of GLGZD-IP group significantly decreased was compared with MCAO group. The mean fluorescence of [Ca^2+^]*_i_* in cortex and striatum neurone of drug-treated group (GLGD-CP, GLGZD-IP, and THF) decreased in numerical order GLGZD-IP < GLGD-CP < THF, compared with MCAO group. Detailed experimentation results are listed in Table 2 and fluorescence micrographs are shown in Figure 2.

**Table 2.** Detection results of [Ca^2+^]*_i_* in cerebral cortex and striatum neurone of MCAO rats (*n* = 6, x¯ ±s, nmol/l).

Groups	Cortex	Striatum
SO control	155.58 ± 6.37	180.51 ± 5.86
MCAO model	574.57 ± 17.53△△	585.43 ± 18.49△△
GLGZD-CP(THF 6 g)	335.78 ± 13.50*	327.21 ± 14.45*
GLGZD-IP(THF 30 g)	268.26 ± 21.33**	247.38 ± 19.12**
THF30 g	516.69 ± 15.41^C^	502.32 ± 16.36^C^

△ *p* ≤ .05 versus SO control.

△△ *p* ≤ .01 versus SO control.

* *p* ≤ .05 versus MCAO model.

** *p* ≤ .01 versus MCAO model.

C *p* > .05 versus MCAO model is NOT different.

### Docking programmes evaluation

A popular eHiTS software package was used for flexible docking. Active site detection was carried out using the “-complex” parameter, docking procedure must be validated with the ligand of the protein target, to choose the RMSD as the main parameter describing docking accuracy. The average value of the RMSD can be heavily influenced by some “off the scale” results, thus we also calculated the number of successfully docked pairs. It is defined as the ratio of pairs for which top score or best pose conformations are below the given threshold in comparison with all evaluated pairs. The RMSD between the values of theoretical calculations with the data of experiments must be below 2.0. The program automatically detected the ligand in the complex and selected the part of target protein within a 7 Å margin around the ligand to be the active site. The compound was then docked into the active site using the highest accuracy mode of docking (“-accuracy” parameter set to 6)^23^.

The statistically derived eHiTS scoring function offers an outstanding binding affinity prediction capability. The score was expressed by pK_d_ (the value of K_d_ or K_i_, and the score is at −6, and the activity is at the μM level). The ligands from PDB bind into three groups according to the strength of binding to the corresponding protein target. The “strong” contained the ligands for which the concentration necessary to inhibit the protein activity was lower than 4.5 × 10^−8^ nM, “medium” which had their pK_d_ between 4.5 × 10^−8^ nM and 3.6 × 10^−6^ μM, and the inhibitors “weak” for which the concentration of the compound to inhibit protein was greater than 3.6 × 10^−6 ^μM^24^. We selected the compound with the best score and speculated the detail binding patterns.

### Importance small molecular compounds affecting target protein/enzyme

A total of 248 compounds were used in the molecular docking studies which the 2D structures were using ChemDraw^®^ Pro 13.0 followed by 3D structure conversion and energy minimisation. Definition of the 4JWX, 3QEL, 4DH0, and 1A29 binding pocket was achieved using the structural information of the NR2A: 1N4, NR2B: ifenprodil, calcineurin_FKBP12: FK506, and calmodulin: trifluoperazine; protein–ligand interaction. Explorations of the interactions of amino acid residues through molecular docking on NR2A, NR2B, FKBP12, and calmodulin, because of the limitation of the article space, listed 248 compounds, of which achieved good eHiTS scores top 10 and dissociate constant were in Table 3.

**Table 3.** Small molecules bound with four targets with the eHiTS score and dissociate constant.

Rat_NR2A			NR2B			Calcineurin_FKBP12			Calmodulin		
Compounds	Score	K_d_ (μM)	Compounds	Score	K_d_(μM)	Compounds	Score	K_d_(μM)	Compounds	Score	K_d_ (μM)
1N4	−12.812	1.54 × 10^−13^	Ifenprodil	−4.885	1.30 × 10^−5^	FK-506	−5.663	2.17 × 10^−6^	Trifluoperazine	−4.324	4.74 × 10^−5^
Benzoylpaeoniflorin	−6.985	1.04 × 10^−7^	Gallotannin	−7.031	9.31 × 10^−8^	1,2,3,4,6-Pentagalloylglucose	−8.299	5.02 × 10^−9^	Glycyrrhizic acid	−7.937	1.17 × 10^−8^
Isopaeoniflorin	−6.857	1.39 × 10^−7^	Strictinin	−6.133	7.36 × 10^−7^	Neowilforine	−7.58	2.63 × 10^−8^	Glyyunnanprosapogenin D	−7.683	2.07 × 10^−8^
Gallotannin	−6.091	8.11 × 10^−7^	Benzoylpaeoniflorin	−6.057	8.77 × 10^−7^	Tellimagrandin I	−7.498	3.18 × 10^−8^	Geraniin	−7.499	3.17 × 10^−8^
Glycyroside	−5.456	3.50 × 10^−6^	Stigmasterol-beta-D-glucoside	−6.031	9.31 × 10^−7^	Pedunculagin	−7.119	7.60 × 10^−8^	Casuarictin	−7.464	3.44 × 10^−8^
Oxypaeoniflorin	−5.281	5.24 × 10^−6^	Isobenzoylpaeoniflorin	−5.988	1.03 × 10^−6^	Licoricesaponine C2	−6.778	1.67 × 10^−7^	Catharanthamine	−7.395	4.03 × 10^−8^
Protocatechuic acid-3-glucoside	−5.267	5.41 × 10^−6^	Liquiritigenin-7-O-beta-D-(3-O-acetyl)-apiofuranosyl-4'-O-beta-D-Glucopyranosid	−5.941	1.14 × 10^−6^	3-Epiursolic acid	−6.534	2.92 × 10^−7^	6''-O-p-Coumaroylgenipingentiobioside	−6.995	1.01 × 10^−7^
Isobenzoylpaeoniflorin	−5.259	5.51 × 10^−6^	6''-O-p-Coumaroylgenipingentiobioside	−5.853	1.40 × 10^−6^	Glycyrrhizic acid	−6.517	3.04 × 10^−7^	Pedunculagin	−6.977	1.05 × 10^−7^
Uralenol-3-methylether	−5.237	5.79 × 10^−6^	Oxypaeoniflorin	−5.801	1.58 × 10^−6^	Stigmasterol-beta-D-glucoside	−6.464	3.43 × 10^−7^	Isopaeoniflorin	−6.786	1.64 × 10^−7^
Hptaphylline	−5.138	7.28 × 10^−6^	1-o-beta-d-glucopyranosylpaeonisuffrone	−5.752	1.77 × 10^−6^	Glyeurysaponin	−6.381	4.16 × 10^−7^	1-o-beta-d-glucopyranosyl-8-o-benzoylpaeonisuffrone	−6.780	1.66 × 10^−7^
Licoricone	−5.128	7.45 × 10^−6^	Albiflorin	−5.745	1.77 × 10^−6^	Casuarictin	−6.303	4.98 × 10^−7^	4-o-methyl-paeoniflorin	−6.482	3.29 × 10^−7^

### Screening assay for inhibitors of NR2A protein

**Figure 3.**
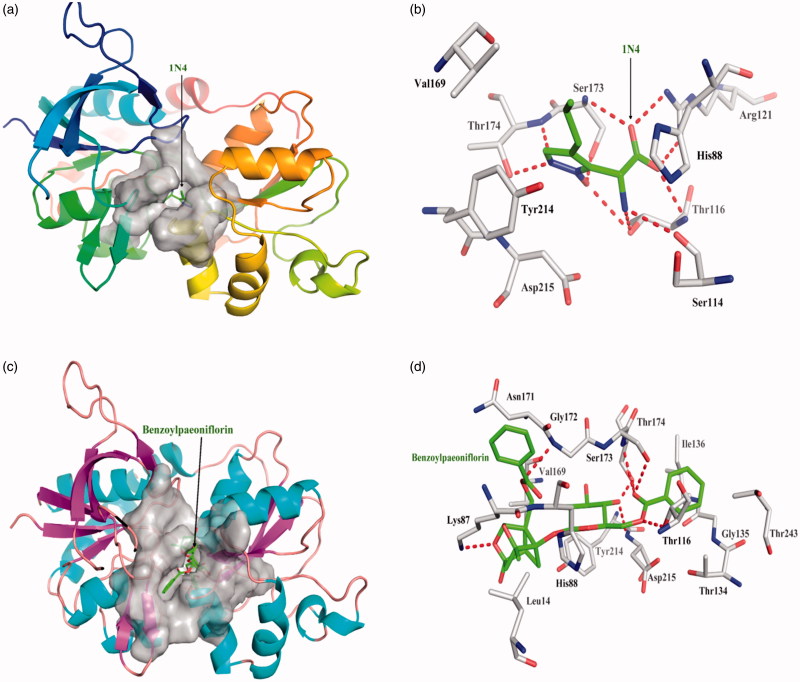
The figures of NR2A /ligand interactions: (a) The positive control binding mode of 1N4 to NR2A protein ribbon, protein was shown in ribbon and 1N4 in green sticks, the active pocket was shown in surface form. (b) 1N4 and NR2A bind schema detail maps, the amino acid residues were labelled in the form of white sticks and 1N4 in green sticks, the red dotted line illustrates the hydrogen bond interaction. (c) The binding mode of Benzoylpaeoniflorin to NR2A protein ribbon. (d) Benzoylpaeoniflorin and NR2A bind schema detail maps; the notation used here were same as positive control.

One acknowledged inhibitor of (2 R)-amino (1-hydroxy-4-propyl-1H-pyrazol- 5-yl) ethanoic acid (1N4) is a strong and selective binder to NR2A protein with dissociate constant 1.54 × 10^−13^. 1N4 interacts closely with NR2A protein by hydrogen bond Thr116, Ser114, Arg121, Ser173, and Thr174, respectively; 1N4 interacted with other amino acid residues mainly exists hydrophobic interaction. Top 10 among 248 compounds were strong and selective binders to NR2A with a dissociate constant (K_d_) values were range of 0.1–1 μM (Table 3). Benzoyl paeoniflorin source from *Paeonia albiflora* exhibits the most affinity intermolecular interactions with the target. The six red dashed lines highlight interacts by Lys87, Thr116, Gly172, Ser173, Thr174, and Asp215 form hydrogen bonds; hydrophobic interactions with other amino acids. Benzoylpaeoniflorin showed slightly lower binding affinity than 1N4 (Figure 3), yet higher the other inhibitor NVP-AAM077.

### Screening assay for inhibitors of NR2B protein

**Figure 4.**
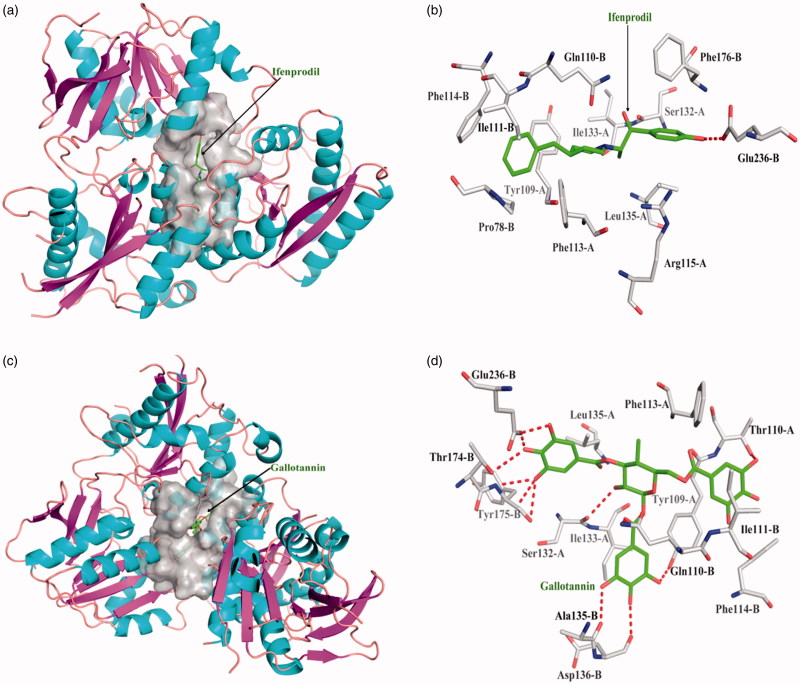
The figures of NR2B/ligand interactions: (a) The positive control binding mode of ifenprodil to NR2B protein ribbon, protein was shown in ribbon and ifenprodil in green sticks, the active pocket was shown in surface form. (b) Ifenprodil and NR2B bind schema detail maps, the amino acid residues were labelled in the form of white sticks and ifenprodil in green sticks, the red dotted line illustrates the hydrogen bond interaction. (c) The binding mode of Gallotannin to NR2B protein ribbon. (d) Gallotannin and NR2B bind schema detail maps; the notation used here were same as positive control.

One acknowledged inhibitor of ifenprodil is selective binder to NR2B protein with dissociate constant 1.30 × 10^−5^. Ifenprodil interacts the NR2B protein with the chain B in Glu236 form one hydrogen bond; ifenprodil interacted with other amino acid residues mainly exists hydrophobic interaction. Top 10 among 248 compounds were strong and selective binders to NR2B with a dissociate constant (K_d_) values were range of 0.01 ∼ 1 μM (Table 3). Gallotannin exhibits the most affinity intermolecular interactions with the target. Gallotannin interact the NR2B protein with the chain A in Ser132 form one hydrogen bond, chain B in Gln110, Ala135, Asp136, Thr174, Tyr175, and Glu236 form six hydrogen bonds; hydrophobic interactions with other amino acids. Gallotannin showed higher binding affinity than ifenprodil (Figure 4).

### Screening assay for inhibitors of FKBP12 enzyme

**Figure 5.**
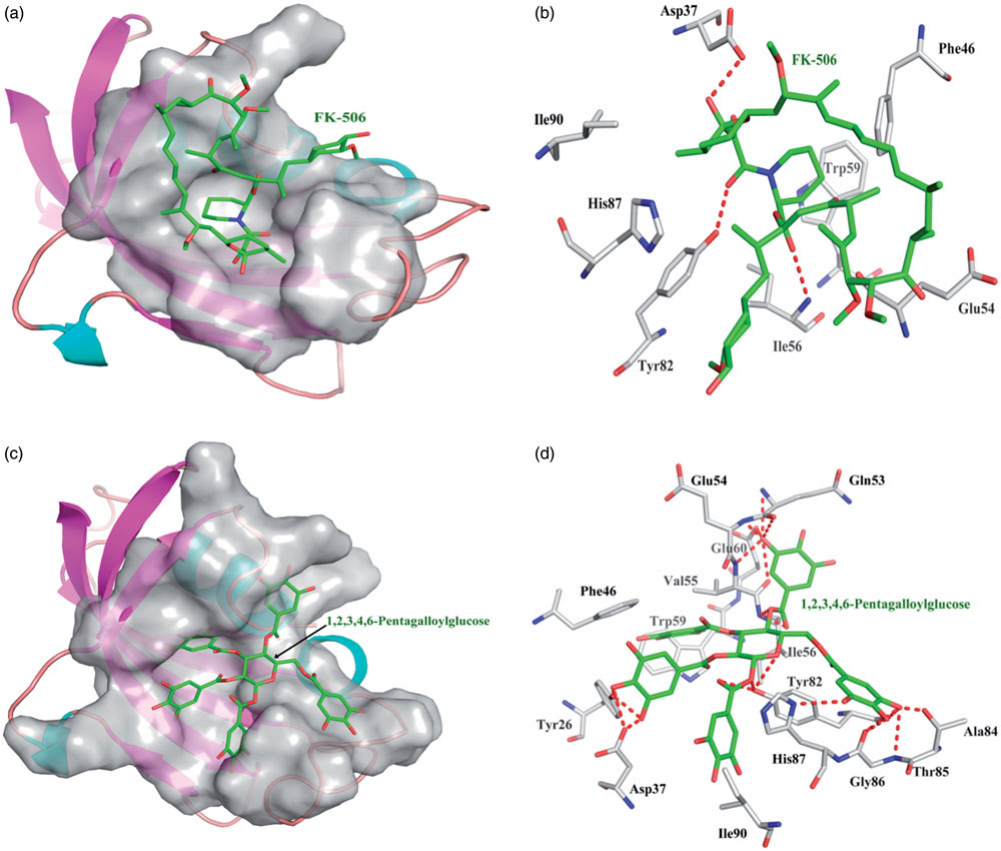
The figures of FKBP12/ligand interactions: (a) The positive control binding mode of FK-506 to FKBP12 protein ribbon, protein was shown in ribbon and FK-506 in green sticks, the active pocket was shown in surface form. (b) FK-506 and FKBP12 bind schema detail maps, the amino acid residues were labelled in the form of white sticks and FK-506 in green sticks, the red dotted line illustrates the hydrogen bond interaction. (c) The binding mode of 1,2,3,4,6-Pentagalloylglucose to FKBP12 protein ribbon. (d) 1,2,3,4,6-Pentagalloylglucose and FKBP12 bind schema detail maps; the notation used here were same as positive control.

One acknowledged enzyme inhibitor of FK-506 is selective binder to FKBP12 enzyme with dissociate constant 2.17 × 10^−6^. FK-506 interacts the FKBP12 enzyme with the Asp37, Ile56, and Tyr82 to form three hydrogen bonds, FK-506 interacted with other amino acid residues mainly exists hydrophobic interaction. Top 10 among 248 compounds were strong and selective binders to FKBP12 with a dissociate constant (K_d_) values were range of 0.001–1 μM (Table 3). 1,2,3,4,6-Pentagalloylglucose exhibits the most affinity intermolecular interactions with the target. 1,2,3,4,6-Pentagalloylglucose interact the FK-506 with Tyr26, Asp37, Gln53, Glu54, Val55, Ile56, Glu60, Tyr82, Ala84, Thr85, Gly86, and His87 to form many hydrogen bonds; hydrophobic interactions with other amino acids. 1,2,3,4,6-Pentagalloylglucose showed higher binding affinity than FK-506 (Figure 5).

### Screening assay for inhibitors of Calnodulin protein

**Figure 6.**
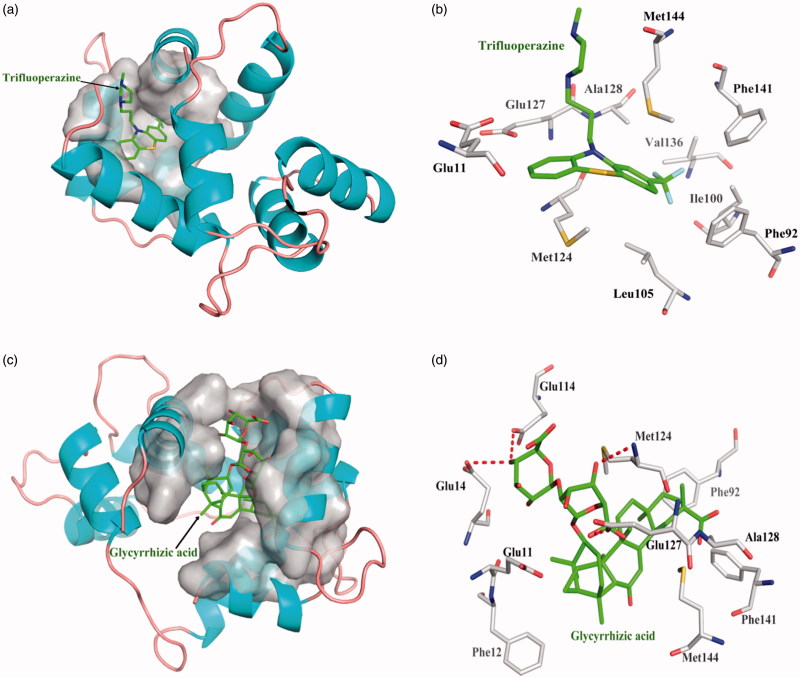
The figures of Calnodulin/ligand interactions: (a) The positive control binding mode of Trifluoperazine to Calnodulin protein ribbon, protein was shown in ribbon and Trifluoperazine in green sticks, the active pocket was shown in surface form. (b) Trifluoperazine and Calnodulin bind schema detail maps, the amino acid residues were labelled in the form of white sticks and Trifluoperazine in green sticks, the red dotted line illustrates the hydrogen bond interaction. (c) The binding mode of Glycyrrhizic acid to Calnodulin protein ribbon. (d) Glycyrrhizic acid and Calnodulin bind schema detail maps; the notation used here were same as positive control.

One acknowledged inhibitor of Trifluoperazine is selective binder to Calnodulin protein with dissociate constant 4.74 × 10^−5^. Trifluoperazine interacts with Calnodulin amino acid residues only hydrophobic interaction, but not forming hydrogen bond. Top 10 among 248 compounds were strong and selective binders to Calnodulin with a dissociate constant (K_d_) values were range of 0.01–0.1 μM (Table 3). Glycyrrhizic acid source from *Glycyrrhiz* exhibits the most affinity intermolecular interactions with the target. The four red dashed lines highlight interacts by Glu14, Glu114, Met124, and Glu127 form hydrogen bonds; hydrophobic interactions with other amino acids. Glycyrrhizic acid showed higher binding affinity than Trifluoperazine (Figure 6).

### Trichosanthin target protein docking

**Figure 7.**
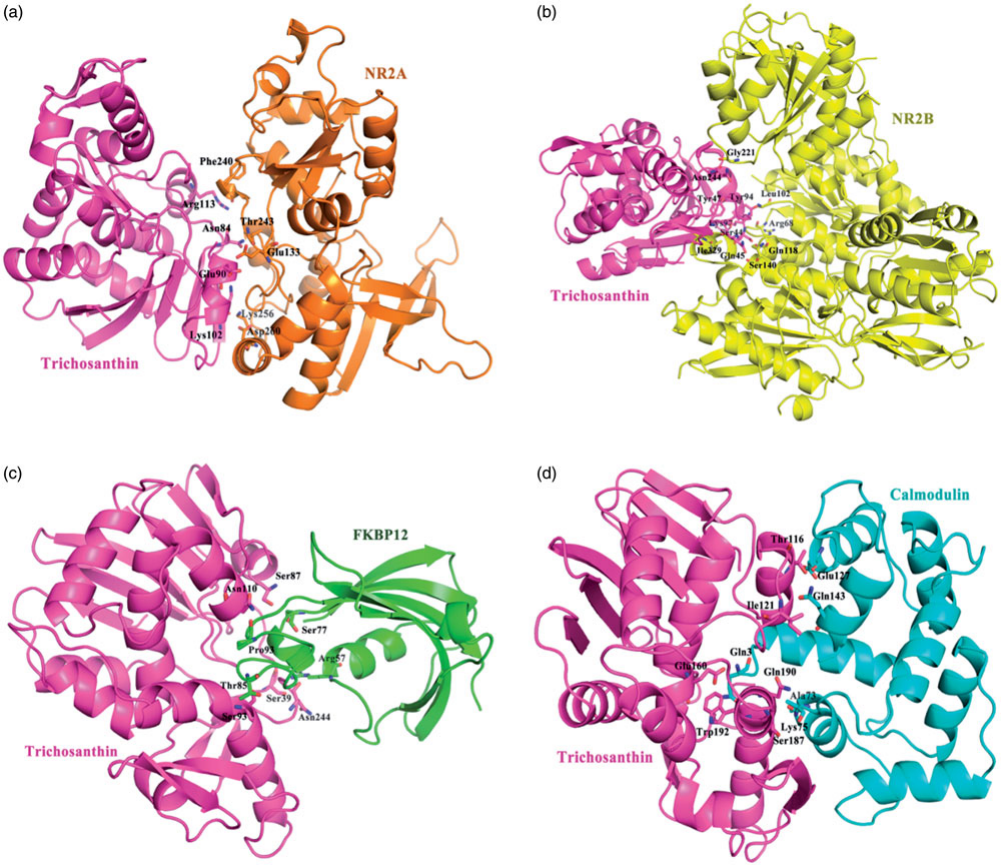
The figures of Trichosanthin was interacted with four target proteins: (a) The binding mode of Trichosanthin to NR2A protein, Trichosanthin protein was shown in violet ribbon and NR2A in orange ribbon; (b) The binding mode of Trichosanthin to NR2B protein, Trichosanthin protein was shown in violet ribbon and NR2B in yellow ribbon; (c) The binding mode of Trichosanthin to FKBP12 enzyme, Trichosanthin protein was shown in violet ribbon and FKBP12 in green ribbon; (d) The binding mode of Trichosanthin to Calnodulin protein, Trichosanthin protein was shown in violet ribbon and Calnodulin in cyan ribbon. The key amino acid residues of the protein-protein or protein–enzyme combinations were labelled in the form of sticks.

The herb Tianhuafen is rich in protein, trichosanthin (TCS). Predicting of protein–protein interaction was carried out ZDOCK software. ZDOCK is an initial-stage docking algorithm that uses a fast Fourier transform (FFT) to find the three-dimensional (3D) structure of a protein complex. The ZDOCK algorithm was optimised by shape complementarity, electrostatics, desolvation free energy, etc. three parameters. Using the default of ZDOCK, TCS Protein Data Bank files (PDB ID 1TCS) was input, the search was performed by randomly perturbing both the receptor and ligand to avoid starting from a near-native state, and then discretizing them into discrete functions for the receptor and ligand, onto separate 3D grids, which result in 3600 total conformations. For ZRank score was implemented for quickly and effectively reranking rigid-body docking predictions. Each conformation, only the top-scoring translation is found by performing a cross-correlation^25^. Protein–protein docking results: The amino acid residues Asn84, Glu90, Lys102, and Arg113 from TCS has been active participation interacted with Glu133, Phe240, Thr243, Lys256, and Asp260 from NR2A, ZRank Score= −78.03; The amino acid residues Ser44, Gln45, Tyr47, Tyr94, Lys97, and Asn244 from TCS has been active participation interacted with Arg68, Leu102, Gln118, Ser140, Gly221, and Ile329 from NR2B, ZRank Score= −88.00; The amino acid residues Ser39, Ser87, Ser93, Asn110, and Asn244 from TCS has been active participation interacted with Arg57, Ser77, Thr85, and Pro93 from FKBP12, ZRank Score= −79.50; The amino acid residues Thr116, Ile121, Glu160, Ser187, Gln190, and Trp192 from TCS has been active participation interacted with Gln3, Ala73, Lys75, Glu127, and Gln143 from Calnodulin, ZRank Score= −102.57 (Figure 7).

## Conclusion

In summary, this work investigated the GLGZD anti intracellular calcium overload in cortex and striatum neurone of MCAO rats. Potential biological active ingredients against NR2A, NR2B, FKBP12, and Calnodulin proteins/enzyme were screened using combination of small molecular docking and protein–protein docking.

GLGZD could decreased the concentration of [Ca^2+^]*_i_* in cortex, striatum in MCAO group, because of increasing the dosage of Tianhuafen from 6 g up to 30 g, GLGZD-IP the effect was better than GLGZD-CP; however, only single herb, Tianhuafen reach dosages of 30 g, failure to respond to medical treatment of calcium overload. This may be the mysteries of Chinese medicine compound prescriptions.

Screening assay for active ingredients of intracellular calcium overload exhibited notable that some small molecular compounds should be possible to develop NR2A and NR2B NMDA receptors antagonists, some inhibit calcineurin after forming complexes with cyto-plasmic binding proteins FKBP12, other compounds disturbed Ca^2+^ interaction with CaM which Ca^2+^ can no longer interact with its target enzymes, it is likely led to inactivation of Ca^2+^-CaM. The protein–protein docking analysis of TCS domain and NR2A, NR2B, FKBP12 and Calnodulin proteins provided insight into the important residues that could form good interactions with the domains of intracellular calcium targets. GLGZD contains Trichosanthin and various small molecular that they are the potential active ingredients, one-component interact with multi-targets or multi-components against multi-targets which “multicomponent systems” is capable to create pharmacological superposition effects. Although these promising compounds need to be further experimentally tested against the four analysed targets and confirmed their efficacy or benefit, using computer virtual screening, the number of actual screening compounds can be reduced and the discovery efficiency of lead compounds can be improved. The Chinese medicine compound prescriptions could be considered as promising sources of potential candidates for discovery new drugs^26^.
